# Novel Roles of cAMP Receptor Protein (CRP) in Regulation of Transport and Metabolism of Carbon Sources

**DOI:** 10.1371/journal.pone.0020081

**Published:** 2011-06-01

**Authors:** Tomohiro Shimada, Nobuyuki Fujita, Kaneyoshi Yamamoto, Akira Ishihama

**Affiliations:** 1 Department of Frontier Bioscience, Hosei University, Koganei, Tokyo, Japan; 2 Micro-Nanotechnology Research Center, Hosei University, Koganei, Tokyo, Japan; 3 Department of Biotechnology, National Institute of Technology and Evaluation, Shibuya-ku, Tokyo, Japan; Baylor College of Medicine, United States of America

## Abstract

CRP (cAMP receptor protein), the global regulator of genes for carbon source utilization in the absence of glucose, is the best-studied prokaryotic transcription factor. A total of 195 target promoters on the *Escherichia coli* genome have been proposed to be under the control of cAMP-bound CRP. Using the newly developed Genomic SELEX screening system of transcription factor-binding sequences, however, we have identified a total of at least 254 CRP-binding sites. Based on their location on the *E. coli* genome, we predict a total of at least 183 novel regulation target operons, altogether with the 195 hitherto known targets, reaching to the minimum of 378 promoters as the regulation targets of cAMP-CRP. All the promoters selected from the newly identified targets and examined by using the *lacZ* reporter assay were found to be under the control of CRP, indicating that the Genomic SELEX screening allowed to identify the CRP targets with high accuracy. Based on the functions of novel target genes, we conclude that CRP plays a key regulatory role in the whole processes from the selective transport of carbon sources, the glycolysis-gluconeogenesis switching to the metabolisms downstream of glycolysis, including tricarboxylic acid (TCA) cycle, pyruvate dehydrogenase (PDH) pathway and aerobic respiration. One unique regulation mode is that a single and the same CRP molecule bound within intergenic regions often regulates both of divergently transcribed operons.

## Introduction

The central metabolism of carbon source catabolism for energy production consists of three pathways, the main glycolysis (Embden-Meyerhof-Parnas; EMP) pathway, pentose-phosphate (PP) pathway and tri-carboxylic-acid (TCA) cycle [1]. These three pathways also provide a number of building blocks for synthesis of a number of components for construction of cell architecture [1]. The catabolic functions and regulations of the individual enzymes involved in these processes have been characterized in details for both prokaryotes and eukaryotes. Expression regulation of the genes coding for these enzymes has been studied in details using the model prokaryote *Escherichia coli* (for reviews see [2]–[4]), raising the concept that CRP (cAMP receptor protein) or CAP (catabolite gene activator protein) is the global regulator controlling transcription of these genes.

CRP was the first purified [5], the first crystallized [6] and the best studied transcription activator from *Escherichia coli* [7]–[13], and plays a key role in activation of a number of the genes for utilization of carbon sources other than glucose (for reviews see 7, [14]–[16]). The CRP regulon includes the genes encoding the transporters and the catabolic enzymes of non-glucose sugars [9], [11]–[13], [15]. Expression of these genes is activated in the absence of glucose through functional conversion of CRP into an active form after interaction with an effector cAMP [17], which is synthesized by the membrane-bound adenylate cyclase. The cyaA gene encoding adenylate cyclase is activated in the absence of glucose [17]. The intracellular level of cAMP is controlled in response to glucose availability [11], [13], [18]. The functional CRP protomer is composed of two molecules of CRP, each being associated with cAMP. The C-terminal domain contains the characteristic helix-turn-helix (H-T-H) motif that is responsible for interaction with CRP-box consisting of a plindromic TGTGAnnnnnnTCACA sequence associated with target promoters [19]. Binding of cAMP to its N-terminal domain leads to activate the C-terminal DNA-binding domain [20], [21]. When cAMP-CRP binds DNA, it induces DNA bending of about 87^O^ [16], [22], [23].

cAMP-CRP is a dual regulator, acting as an activator or a repressor [13], [18]. The mode of transcription regulation by cAMP-CRP correlates with the position of DNA binding relative to the target promoter [24], [25]. CRP was the first transcription regulator that was identified to interact directly with the promoter-bound RNA polymerase for function [24]–[26]. Up to the present time, a total of 195 target promoters have been recorded in Regulon DB [27], including a large number of the genes encoding transporters and catabolic enzymes of sugars other than glucose (note that some of the regulation targets were predicted based on the presence of CRP-binding sequences).

As a short-cut approach for identification of the whole set of genes under the control of a given transcription factor (TF), we developed an improved “Genetic SELEX” screening system [28]. Initially we cloned and sequenced TF-associated genome DNA fragments for mapping on the *E. coli* genome, but later improved by employing DNA tilling array for mapping of SELEX fragments on the *E. coli* genome [29], [30]. After Genomic SELEX screening of Cra, originally identified as the repressor FruR but later renamed to Cra (catabolite repressor and activator) [31], its regulation targets suddenly increased more than 8-fold to 178 [30], the number as many as that of CRP targets. We then proposed a novel model that Cra is another global regulator of the genes for carbon metabolism, playing a role in balancing the level of enzymes involved in glycolysis. This finding raises the need for reevaluation of the differential roles of two global regulators, CRP and Cra, in the overall regulation network of carbon source metabolism.

In this study, we performed a systematic search for the whole set of regulation targets of cAMP-CRP by using the Genomic SELEX screening system. Using the collection of 254 different CRP-binding sequences thus identified, a total of at least 183 promoters have been newly identified as the regulation targets of CRP. By using the LacZ reporter assay, some of these novel CRP target promoters were confirmed to be under the control of CRP in vivo. Including the hitherto identified targets, the total number of operons under the direct control of cAMP-CRP was estimated to range from minimum 378 to maximum more than 500. After analysis of the functions of newly identified regulation target operons, we propose some novel regulatory roles and the regulation modes of CRP.

## Materials and Methods

### Bacterial strains and plasmids

*Escherichia coli* BW25113 (W3110 *lacI*q *rrnBT14 Insert > symbol D*Δ*lacZWJ16 hsdR514* Δ*araBADAH33* Δ*rhaBADLD78*), and *crp* disruptant JW5702 were the products of the Keio collection [32] and obtained from the *E. coli* Stock Center (National Institute of Genetics, Mishima, Japan). Cells were grown in LB medium at 37°C under aeration with constant shaking at 140 rpm. Cell growth was monitored by measuring the turbidity at 600 nm. Plasmid pRS551 was used for the reporter assay of CRP-depending promoters (see below). For maintenance of pRS551, ampicilin and kanamycin were added each at a final concentration of 50 mg/ml.

### Purification of CRP protein

*E. coli* BL21(DE3) transformant with the CRP expression plasmid pCRP encoding His-tagged CRP was grown in LB broth, and protein expression was induced by adding 1 mM IPTG. After 3 h of induction, cells were harvested and subjected to CRP purification. Purification of His-tagged CRP was performed according to the standard procedure [33], [34]. In brief, lysozyme-treated cells were sonicated in the presence of 100 mM phenylmethylsulfonyl fluoride. After centrifugation of cell lysate (30 ml) at 15,000 rpm for 60 min at 4°C, the resulting supernatant was mixed with 2 ml of 50% Ni-nitrilotriacetic acid (Ni-NTA) agarose solution (Qiagen) and loaded onto a column. After being washed with 10 ml of lysis buffer, the column was washed with 10 ml of washing buffer (50 mM Tris-HCl, pH 8.0, at 4°C, 100 mM NaCl). Proteins were then eluted with 2 ml of an elution buffer (200 mM imidazole, 50 mM Tris-HCl, pH 8.0, at 4°C, 100 mM NaCl) and dialyzed against a storage buffer (50 mM Tris-HCl, pH 7.6, 200 mM KCl, 10 mM MgCl2, 0.1 mM EDTA, 1 mM dithiothreitol, and 50% glycerol). CRP used throughout this study was >95% pure as judged by sodium dodecyl sulfate-polyacrylamide gel electrophoresis (SDS-PAGE).

### Genomic SELEX search for the CRP-binding sequences

The genomic SELEX method was carried out as previously described [28]. A mixture of DNA fragments of the *E. coli* K-12 W3110 genome was prepared after sonication of purified genome DNA, and cloned into a multi-copy plasmid pBR322. In each SELEX screening, the DNA mixture was regenerated by PCR. For SELEX screening, 5 pmol of the mixture of DNA fragments and 10 pmol each of His-tagged CRP were mixed in a binding buffer (10 mM Tris-HCl, pH 7.8 at 4°C, 3 mM magnesium acetate, 150 mM NaCl, and 1.25 mg/ml bovine serum albumin) and incubated for 30 min at 37°C. For the effector(+) reaction of CRP, 10 insert>symbol mµM adenosine 2′,3′-cyclic monophosphate (cAMP) (SIGMA) was added during the binding reaction and also subsequent isolation procedure of CRP-DNA complexes. The DNA-transcription factor mixture was applied to a Ni-NTA column, and after washing out unbound DNA with the binding buffer containing 10 mM imidazole, DNA-protein complexes were eluted with an elution buffer containing 200 mM imidazole. DNA fragments recovered from the complexes were PCR-amplified.

For SELEX-clos (cloning-sequencing) analysis, PCR products were cloned into pT7 Blue-T vector (Novagen) and transformed into *E. coli* DH5a. Sequencing of each clone was carried out using the T7 primer (5′-TAATACGACTCACTATAGGG-3′). For SELEX-chip (DNA tilling-array chip) analysis, PCR-amplified products of the isolated DNA-protein complexes obtained in the presence or absence of the effecter were labeled with Cy5, and mixed with the original mixture of genome DNA fragments labeled with Cy3. The fluorescent labaled DNA mixtures were hybridized to a DNA microarray consisting of 43,450 species of 60 b-long DNA probe, which are designed to cover the entire *E. coli* genome at 105 bp interval (Oxford Gene Technology, Oxford, UK). The Cy5 fluorescent intensity of test sample at each probe was normalized with that of the corresponding peak of Cy3 fluorescence of the original library. The Cy5/Cy3 ratio was measured and plotted along the *E. coli* genome.

### Reporter assay of the promoter activity and regulation

To measure the activity and regulation of promoters, a LacZ-reporter assay was employed using pRS551 plasmid vector [35]. Promoter fragments of approximately 500 bp in length between the initiation codon and 500 bp upstream sequence were amplified by PCR using a pair of primers (F and R) (for sequences see Table S1). The fragments were digested with *Bam*HI and *Eco*RI, and then ligated to pRS551. Construction of the plasmids was confirmed by DNA sequencing. Plasmids were transformed into test *E. coli* strains, wild-type BW25113, crp mutant JW5702. Overnight cultures in LB medium were diluted 1∶1000 into fresh medium, and cells were grown for 4 hrs to an OD_600_ of 0.4–0.5. The activity of β-galactosidase was measured according to the standard Miller method [36]. The measurement was performed four times to get the average values.

## Results

### Search for the regulation targets of CRP by using Genomic SELEX screening: SELEX-clos analysis

In order to get insight into the genome regulation by CRP for control of carbon source utilization in *E. coli*, we first tried to identify the whole set of target promoters, genes and operons under the control of CRP. For this purpose, we employed ‘Genomic SELEX’ screening, in which DNA fragments carrying the recognition sequence by CRP were isolated as CRP-DNA complexes from a mixture of genome DNA fragments (28). For screening of DNA sequences with affinity to CRP, purified His-tagged CRP was mixed with a collection of *E. coli* genome fragments of 200–300 bp in length, and CRP-bound DNA fragments were affinity-purified. Since CRP exhibits functional inter-conversion depending on the presence or absence of specific effector cAMP [11], we carried out Genomic SELEX in the presence and absence of cAMP. The original substrate mixture of genomic DNA fragments used for the Genomic SELEX screening formed smear bands on PAGE. After repeating the Genomic SELEX cycle in the continuous presence of cAMP, DNA fragments with affinity to CRP were enriched, leading to form sharp PAGE bands. In contrast, the gel pattern of DNA fragments isolated after Genomic SELEX in the absence of cAMP did not change from the original DNA mixture, indicating that little enrichment of specific DNA fragments.

For identification of the sequences of DNA fragments bound to CRP, we first performed SELEX-clos (cloning-sequencing) analysis. A total of 160 SELEX fragments were cloned and sequenced, which were classified into 29 CRP-binding sequences, including 13 previously identified loci and 16 newly identified sites (Table S2). When the CRP-binding sequences are located upstream or near promoters of the flanking genes, these genes are predicted to be under the control of CRP. A number of genes encoding transporters were included in the collection of SELEX-clos clones (see Table S2), including transporters of not only sugars such as maltose (*malK*), fucose (*fucP*), and mannitol (*mtlA*) but also amino acids and nucleosides such as alanine (*cycA*), serine (*dsdX*) and uridine (*nupC*).

In the case of cAMP-CRP, the binding sites are mostly located in intergenic spacer regions between divergently transcribed genes. As an initial screening, we predicated that only of the divergent promoters is under the control of cAMP-CRP (however, note that both promoters are often regulated by cAMP-CRP bound within intergenic regions). For instance, the most abundant 59 clones carried the intergenic sequence between the divergently transcribed yecJ and yecR, of which one is counted as a CRP target (Table S2). Likewise, the collection of next abundant 38 clones carried the intergenic sequence between *yibI* and *mltA*, both being located downstream of the cAMP-CRP binding sequence, of which the *mtlA* operon encoding mannitol-specific PTS permease is known to be activated by cAMP-CRP [37]. SELEX-clos is useful to get information on the order of binding affinity between regulation targets by test DNA-binding transcription factors because the number of isolated DNA sequences by SELEX-clos correlates with the affinity to the test factor (28, 38). SELEX-clos is dominated by high affinity sites, consequently, a massive number of sites would have to be sequenced in order to identify the many weaker low abundant sites.

### Search for the regulation targets of CRP by using Genomic SELEX screening: SELEX-chip analysis

**Figure 1:**
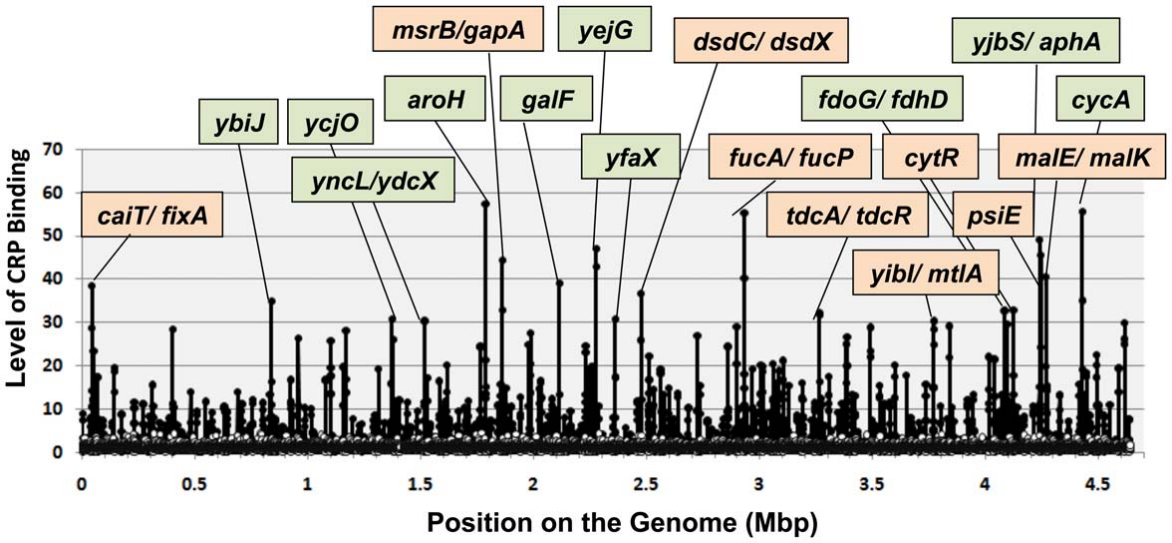
Identification of cAMP-CRP-binding sites on the *E. coli* genome. Genomic SELEX search of cAMP-CRP-binding sequences was performed using the standard procedure [28] in the presence (filled circles) and absence of cAMP (open circles). SELEX fragments were subjected to SELEX-chip analysis using the tilling DNA microarray as described previously (39). Regulation target genes of CRP were predicted from the location of CRP-binding sites (for details see text). The genes associated with the peaks at the cut-off level above 30 are indicated. When the CRP-binding site is located upstream of divergently transcribed genes, two genes are shown, being connected with a flash mark. Genes under light green background represent newly identified CRP targets while those under pale orange background represent the CRP targets listed in Regulon DB [27]. The level of CRP binding (Y-axis) represents the fluorescent intensity ratio between SELEX samples in the presence and absence of CRP.

For identification of the whole set of binding sequences by CRP, we then subjected the mixture of SELEX fragments to the newly developed DNA tilling array analysis (SELEX-chip) [29], [30]. Genomic SELEX fragments obtained with or without the effector cAMP were labeled with Cy5 while the original DNA library was labeled with Cy3. Two sets of the mixture of fluorescent-labeled samples were hybridized separately to the DNA tilling microarray (Oxford Gene Technology, Oxford, UK) [39]. For elimination of the bias of library DNA, the ratio of fluorescence intensity bound to each probe between the test sample and the original library DNA was measured, and plotted against the corresponding position along the *E. coli* genome (Fig. 1). On the DNA tilling array used, the 60 b-long probes are aligned along the *E. coli* genome at 105 bp-intervals, and therefore approximately 300 bp-long SELEX fragments should bind to two or more consecutive probes. This criterion was employed for identification of positive peaks of CRP binding (or elimination of false-positive peaks).

By setting the cut-off level of intensity ratio at 30, a total of 19 high-level peaks with strong affinity to cAMP-CRP were identified (Fig. 1) while the number of peaks increased to 50 by decreasing the cut-off level to 20. The number of CRP-binding peaks further increased to 277 by setting the cut-off level of 4.0. In the absence of cAMP, no specific peaks were identified for CRP (Fig. 1, open circles; but note that open circles are hidden behind closed circles), indicating that the peaks thus identified represent specific binding of cAMP-bound CRP. Of the total of 277 CRP-binding peaks, 258 peak sequences (93%) were found to locate upstream of genes (Table 1; for details of the mapping see Table S3), either upstream of one specific gene or upstream of divergently aligned genes. The rest of 19 peaks (7%) are located downstream of genes. The cAMP-CRP-binding sequences identified by SELEX-clos (Table S2) were all included in the list of cAMP-CRP-binding loci identified by SELEX-chip (Table S3). The cAMP-dependent binding of CRP to some of these target sites was confirmed by PAGE gel shift assay (data not shown).

**Table 1:** Known regulation targets of cAMP-CRP.

A. Targets identified by Genomic SELEX (total, 92)
*araB, araC, araE, araF, ascF, aspA, caiF, caiT, cdd, cpdB, csiD, cstA, cytR, dadA, dctA, dgsA, deoC, dsdX, dusB, epd, exuT, fadL, fixA, flhD, fucA, fucP, galP, gapA, gatY, galS, gcd, gdhA, glgS, glnL glpA, glpT, gltA, gntK, gntP, gntT, guaB, hpt, hyfA, idnD, idnK, isrB, ivbL, kbaZ, lacZ, lsrA, malE, malI, malK, malS, malT, malX, manX, maoC, mglB, mtlA, nagB, nagE, nanC, nmpC, nupC, nupG, ompF, paaA, ppiA, proP, psiE, ptsG, ptsH, putP, raiA, rhaB, rhaS, rpoH, sdhC, serA, serC, tdcA, treB, tsx, udp, uhpT, uidA, ulaA, uxaC, uxuA, xseA, yeiT*

B. Targets not identified by Genomic SELEX (total, 103)
*aceB, acnA, acnB, acs, aer, agp, aldA, aldB, ansB, araJ, argG, bglG, chbB, cirA, crp, csgD, csiE, cyaA, cyaR, cyoA, dcuB, dcuR, dsdA, ebgA, entC, fadD, feaR, fecA, fepA, fiu, focA, fumA, fur, gadA, gadB, gadE, galE, glcC, glgC, glnA, glpD, glpE, glpF, gltB, gntX, grpE, gyrA, hlyE, hofM, hupA, hupB, isrB, lpd, marR, mdh, melA, melR, metK, metY, mhpA, modA, mpl, murQ, nanA, nirB, nrdA, ompA, ompR, osmY, oxyR, pdhR, pncB, ppdD, prpB, prpR, rbsD, relA, rrhaT, rpoS, sbmC, sdhD, sfsA, sodA, sodB, sohB, speC, spf, srlA, tnaC, trg, trxA, ubiG, ugpB, uxaB, uxuR, xylA, xylF, ychH, yfiD, yhfA, yiaJ, yiaK, ykgR, zraS*

The regulation targets of cAMP-CRP listed in RegulonDB [27] are classified into two groups. (A) A total of 91 targets identified by SELEX-chip; and (B) a total of 104 targets not identified by SELEX-chip.

CRP is the best-studied global regulator of *E. coli*, and a total of 195 genes or operons are listed as the regulation targets of cAMP-CRP in *E. coli* databases such as Regulon DB (27) (Table 1). Among the hitherto identified targets, 92 were included in the list of targets identified by Genomic SELEX (Table 1, group-A; for details see Table S3). Except for these 92 known targets, the rest of 183 targets were newly identified by SELEX-chip in this study. Even though a total of 103 known CRP targets were not included in the SELEX-chip list of CRP-binding sites (Table 1, group-B), it is noteworthy that the targets were identified from the SELEX-chip pattern by setting the high cut-off level to avoid background noises (see above). In addition, it should be noted that a number of the known CRP targets in Regulon DB were predicted, without experimental evidence, simply after search of the CRP-box sequence (for details see Discussion).

The locations of CRP-binding sites relative to the gene or operon organization around these newly identified CRP-binding sites are summarized in Table 2. When the CRP-binding site is located upstream of a single gene or operon (and downstream of another neighboring gene), we predicted these downstream genes or operons as regulation targets of CRP (Table 2, group-A). In *E. coli*, transcription factors bound within an intergenic region between divergently organized genes or operons generally regulate transcription toward one direction. A group of CRP-binding sites are located between divergently organized genes or operons (Table 2, group-B). For this group of CRP-binding loci, we tentatively counted only one of the divergently transcribed genes or operons as the regulation target (Table 2, group-B1 and group-B2). In the case of group-B1 (50 CRP-binding sites), CRP binds to non-coding intergenic regions while in the case of group-B2 (14 CRP-binding sites), CRP binds on to open reading frames near the boundary with intergenic spacers. In both group-B1 and group–B2 cases, we tentatively estimated that CRP regulates one of the divergently transcribed genes or operons. However, we noticed such a unique feature of CRP regulation that CRP bound within an intergenic region often regulates both of the divergently transcribed genes or operons (see below). Thus, it can not be excluded yet that for some of group-B1 and –B2 cases, both of the divergently transcribed operons are regulated by cAMP-CRP bound within intergenic regions. Within a total of 195 hitherto identified CRP targets in Regulon DB, the binding sites of 63 targets are located within intergenic regions of divergently transcribed operons (Table 2, goup-C). In these cases, it is not ruled out that CRP regulates transcription toward opposite directions.

**Table 2:** Novel regulation targets of cAMP-CRP on the *E. coli* genome.

A. Newly identified targets (total, 119)
amn, argT, aroM, atpI*, cmtB*, cobU, creD, cycA, deaD, dppA, eco, emrB, eptB, eutS, fadH, fbaB, frlA, fruB*, fryB, galF, gcvT, glk*, gltI, hchA, hda, hemB, hslU, kdgT, kdpE, maa, malZ, moaA, mraZ, mscS, murD, narZ, nepI, nrdD, nuoA*, otsB, pck*, pgi*, pheP, polB, pphB, ppiB, ppsA*, pscK, purM, rihB, rmf, rnk, rplT, rppH, rrfA, rrfB, rrfD, rrfE, rrfG, rrfH, rsmB, setB*, spr, talA*, tppB, tyrT, ubiC, udk,uspF, xapA, valV, visC, wbbJ, xylE, yahL, ybfM, ybhH, ybiH, ybiI, ybiJ, ybiV, ycdZ, yciS, ycjN, ydbJ, ydcC, ydeA, ydeN, ydfZ, ydhP, ydiH, ydjF, ydjN, ydiS, yeaQ, yeaV, yeeR, yehP, yejG, yfaX, yffQ, yffS, yfgM, yfjT, ygfK, yghG, ygiS, yhaO, yhcN, yidK, yjhI, ykfA, ynaJ, yneE, ynfF, yobF, yqgA, yqiI, ysaB

B1. Prediicted targets (divergently transcribed gene sets) (total, 50)
*aaeX<>aaeR, abgA<>abgR, adhE<>ychE, allD<>fdrA, aqpZ<>ybjD, cspD<>clpS, dacD<>sbcB, ddlA<>iraP, dsrA<>yedP, dusC<>yohJ, elbB<>ryhA, fdoG<>fdhD, garP<>garD, gcvA<>gcvB, glyA<>hmp, insG<>yjhB, mipA<>yeaG, mltA<>metZ, ppa<>ytfQ, ptsA<>frwC, puuA<>puuD, rhtA<>ompX, rpiR<>rpiB, wza<>yegH, yagT<>yagU, ybgH<>ybgI, ybiS<>ybiT, ybjJ<>ybjK, ycaC<>ycaD, ycfQ<>bhsA, yciC<>ompW, yehU<>mlrA, ygbI<>ygbJ, ygcW<>yqcE, ygeV<>ygeW, ygfT<>ygfU, yhjA<>treF, yicO<>ade, yieP<>rrsC, yihU<>yihV, yjbS<>aphA, yjcB<>yjcC, yjeH<>groS, yjfO<>yjfP, yjfY<>rpsF, yjiY<>tsr, ykgM<>insE, ymfE<>lit, yncL<>ydcX, ytfJ<>ytfK*

B2. Predicted targets (divergently transcribed gene sets with CRP sites on ORF of spacer genes) (total, 14)
*azoR<hrpA>ydcF, csrB<syd>queF, garP<garD>sohA, kduI<yqeF>yqeG, lpxH<ppiB>cysB, lysP<yeiE>yeiH, matA<matB>matC, minD<minC>ycgJ, rplM<yhcM>yhcB, uraA<upp>purM, yebA<znuA>znuC, yeaZ<yoaA>yoaB, yedJ<yedR>xseX, yqfF<argP>scpA*

C. Predicted targets (genes transcribed toward opposite directly from known target gene) (total, 63)
*acnB, acs, aer, araF, ascF, aspA, cpdB, csgD, cyaA, dadA, deoC, dsdX, entC, fadL, feaR, fepA, fumA, gapA, gdhA, glcC, glgS, glnA, glnL, glpF, gltB, gntX, grpE, hlyE, hofM, ivbL, kbaZ, tsrA, malS, malT, manX, marR, mdh, mhpA, mtlA, murQ, nanC, nmpC, nrdA, nupC, ompR, oxyR, pdhR, pncB, ppiA, putP, serA, sohB, speC, srlA, tdcA, treB, trxA, udp, ulaA, ychH, yfiD, ykgR, zraS*

Overall the total number of regulation target genes or operons of cAMP-CRP could be minimum 378 (183 novel targets including 119 group-A, 50 group-B1, and 14 group-B2; plus 195 known targets). The number of CRP targets should be more because: 1) both of the divergently transcribed genes or operons might be regulated for some of group-B and group-C cases and even for some known targets; and 2) regulation targets increase if the cut-off level of SELEX-chip is set at lower levels [note that only about half of the known CRP targets were picked up at the cut-off level herein employed].

### Consensus recognition sequences of CRP

**Figure 2:**
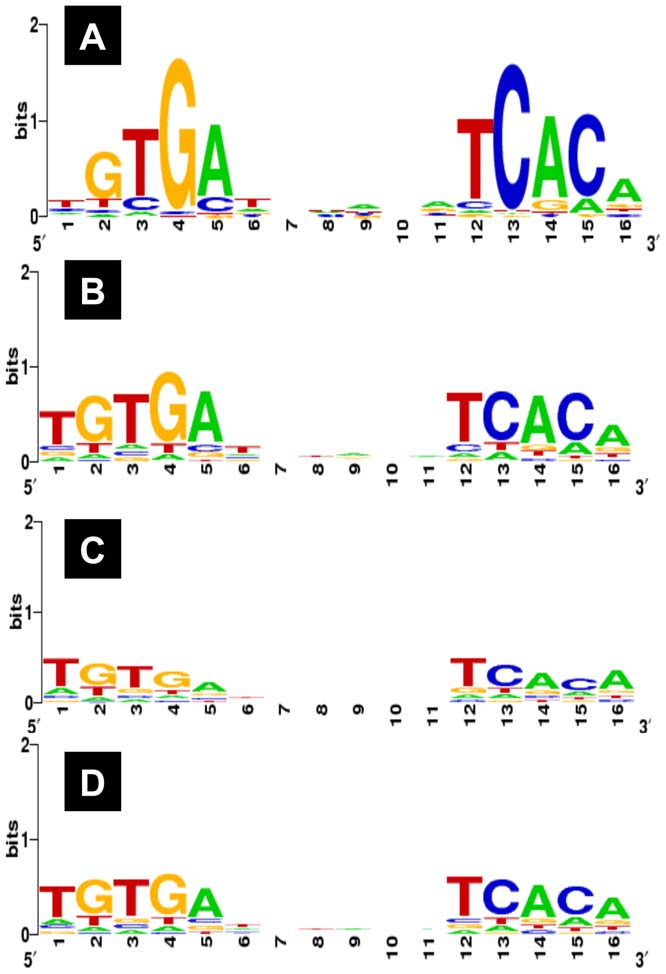
The consensus sequences of CRP binding. The binding sequences of cAMP-CRP were subjected to the Logo analysis for determinatin of the consensus sequences for the following samples: (A) the whole set of CRP targets (total 323 sequences) identified by Genomic SELEX screening in this study; (B) the set of CRP targets (165 sequences) that are listed in Regulon DB and identified by Genomic SELEX; (C) the set of CRP targets (129 sequences) that are listed in Regulon DB but not identified by Genomic SELEX; (D) the whole set of CRP targets (294 sequences) listed in Regulon DB.

The consensus recognition sequences of CRP have been proposed after sequence analysis of the hitherto identified targets (for a review see [38]). As a test of the accuracy of Genomic SELEX screening for cAMP-CRP binding sequences, we searched for the CRP-box sequence using the whole set of 323 CRP-binding sequences from a total of 275 CRP targets (183 novel plus 92 known targets) identified in this study [note that some of the CRP targets carry multiple CRP-box sequences] (for details see Table S4). A collection of 500-bp sequences centered on each peak and using BioProspector (http://ai.stanford.edu/~xsliu/BioProspector/), which was successfully employed for identification of RutR box sequence [33], we identified the 16-bp sequence CRP-box motif for all 323 CRP-binding sites (Fig. 2A). The CRP box-consensus sequence, 5′-TGTGA-N6-TCACA-3′, agrees well with the hitherto identified [38], but indicates clearly that G at position 4 and C at position 13 of the CRP-box are highly conserved in good agreement of their key roles to exhibit the high affinity to CRP. In the Genomic SELEX screening herewith employed, a total of 92 known targets (47%) were identified from a total of 195 CRP targets deposited in Regulon RD. After separate analysis of CRP-box sequence for two groups of CRP targets from Regulon DB, it turned clear that the consensus CRP-box sequence is highly conserved for the CRP targets identified by Genomic SELEX (Fig. 2B). In contrast, the level of CRP-box sequence conservation is significantly lower for the group of unidentified CRP targets (Fig. 2C). This finding supports the usefulness of Genomic SELEX screening for quick identification of the whole set of regulation targets by cAMP-CRP with high accuracy.

### Location of the CRP-binding sites

**Figure 3:**
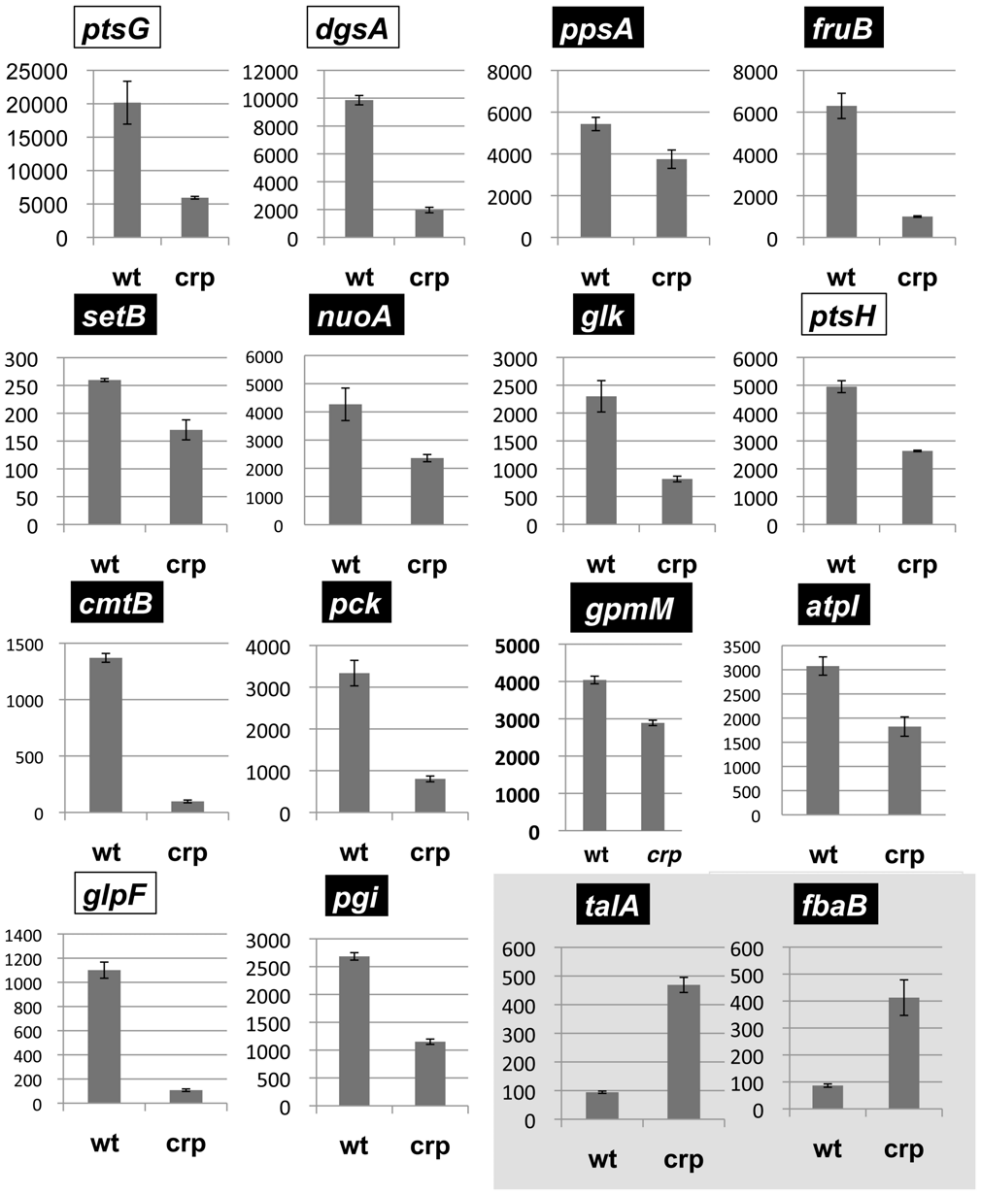
Analysis of promoter regulation *in vivo* by CRP using LacZ fusion. The predicted CRP-target promoters were inserted into the *lacZ* expression plasmid to construct a collection of promoter-*lacZ* fusions. The promoter-dependent expression of LacZ was determined in both wild-type *E. coli* and *crp*-defective mutants (Y-axis, β-galactosidase activity in Miller unit). Except for the *talA and fbaB* promoters (shown under gray background), the activity of all other promoters decreased in the absence of activator CRP, indicating the involvement of CRP as an activator. The newly identified promoters are shown under black background. As references, some known CRP target promoters such as *ptsG*, *dgsA*, *ptsH* and *glpF* (shown under white background) were examined in parallel under the same conditions.

CRP has been identified to regulate some of the enzymes for central carbon metabolism, including *fbaA*, *gapA*, and *pgk*. In addition, we identified in this study the binding of cAMP-CRP to the genes encoding *glk* (glucokinase), *fruK* (1-phosphofructokinase), *yggF* (type II fructose-1,6-bisphosphatase), *ppsA* (phosphoenolpyruvate synthase), *talA* (transaldolase) and tktB (transketolase II). In order to further confirm the accuracy of target prediction by the Genomic SELEX screening, we performed the *lacZ* promoter assay for some of the newly identified target promoters, together with some known targets, in both wild-type and *crp* deletion mutant. Fig. 3 shows the activity of 12 representative promoters that were predicted to be under the control cAMP-CRP after SELEX-chip analysis, together with 4 known promoters, *ptsG* (glucose-specific PTS), *dgsA* (global regulator of carbon metabolism), *ptsH* (PTS Hpr component) and glpF (glycerol facilitator), that are activated by cAMP-CRP. All the putative target genes examined were indeed found to be under the control of CRP, classified in group-A in Table 2, supporting the prediction that the Genomic SELEX screening allows the identification of regulation targets of cAMP-CRP with high accuracy.

Based on the promoter assay, we can also predict the mode of regulation *in vivo*, either activation or repression, for the novel targets. Two promoters, *talA* (transaldolase) and *fbaB* (fructose bisphosphate aldolase), showed increased activity in the *crp*-disrupted mutant, indicating that these two promoters are repressed by cAMP-CRP. All other promoters tested showed decreased activity in the *crp* mutant, including *pps* (phosphoenolpyruvate synthetase), *fruB* (fructose-specific PTS), *setB* (lactose/glucose efflux system), *nuoA* (NADH:ubiquinone oxidoreductase), *glk* (glucokinase), *cmtB* (mannitol-specific PTS), *pck* (phosphoenolpyruvate carboxykinase), *gpmM* (phosphoglycerate mutase), *atpI* (ATP synthase) and *pgi* (glucosephosphate isomerase). For this group of promoters, cAMP-CRP is involved as an activator. The binding sites of activator CRP were mostly located upstream from the promoters while those of repressor CRP were located downstream from the target promoters (Fig. S1) in agreement with the initial mapping of CRP-binding sites on various CRP-regulated promoters [24], [25]. The binding sites of activator CRP are located upstream of the respective promoters, showing a clear periodical pattern of about 10 bp spacing with peak positions starting from −40 (data not shown), in agreement with the direct protein-protein contact model of upstream bound CRP and RNA polymerase on the same DNA surface [40]–[42]. In contrast, the binding sites of repressor CRP overlap with the target promoters or downstream from the promoters.

### Regulation of divergently transcribed genes by CRP

**Figure 4:**
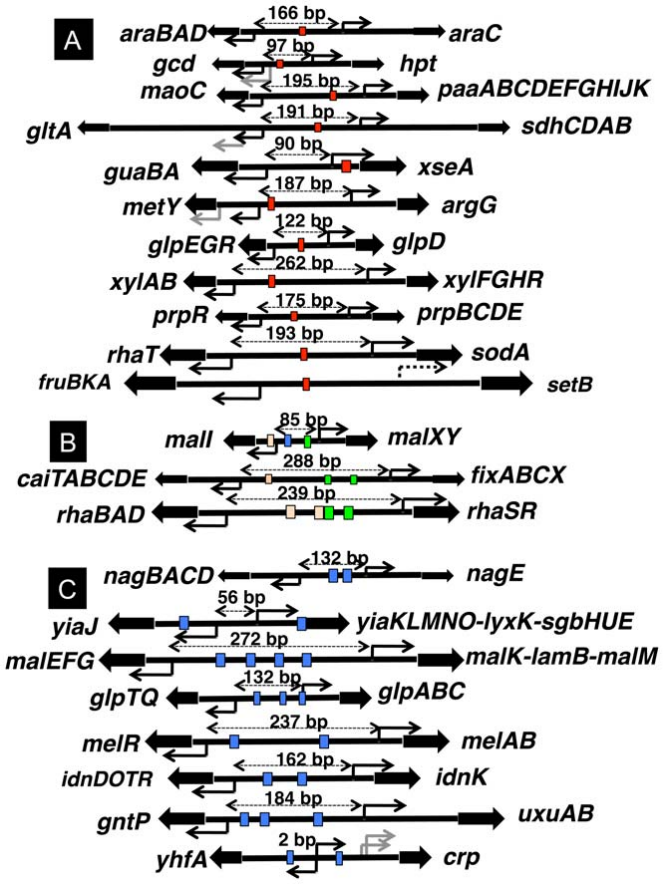
Regulation of the divergently transcribed promoters by CRP. Some of the divergently aligned promoters are controlled by CRP molecule(s) bound within spacers. The gene organization and the binding sites of CRP within the spacer regions are shown: (A) divergent promoters are regulated by single and the same CRP bound within spacers; (B) divergent promoters are regulated by different CRP molecules bound within spacers; and (C) multiple CRP molecules bind to spacer between divergent promoters, but the role of each CRP remains unidentified. The numbers above DNA indicate the distance between the initiation sites of divergent transcription.

In *E. coli*, it is rare that a single and the same regulator controls divergently aligned promoters, which direct transcription toward opposite directions. After a systematic comparison of the CRP-binding sites within intergenic regions of divergent promoters, both being under the control of cAMP-CRP, a single CRP-binding site was detected for a total of 11 cases, *araBAD-araC*, *gcd-hpt*, *maoC-paaABCDEFGHIJK*, *gltA-sdhCDAB*, *guaBA-xseA*, *metY-argG*, *glpEGR-glpD*, *xylAB-xylFGHR*, *prpR-prpBCDE*, *rhaT-sodA* and *fruBKA-setB* (Fig. 4A). One unique feature of the regulation mode by cAMP-CRP is that a single and the same transcription factor bound within an intergenic spacer region controls both of the divergently aligned promoters. Using a model system, El-Robh and Busby [43] performed a detailed study of transcription activation of divergent promoters by a single CRP molecule.

Except for the above 11 cases, there are a number of the divergent promoters, both being apparently under the control of cAMP-CRP. Detailed analysis, however, indicated the presence of multiple CRP-binding sites within intergenic spacers. In three cases including *caiTABCDE-fixABCX*, *malI-malXY* and *rhaBAD-rhaSR*, however, the divergent promoters are regulated by different CRP molecules (Fig. 4B). For other 8 pairs including *nagBACD-nagE*, *yiaJ-yiaKLMN0lyxKsgbHUE*, *malEFG-malKlamBmalM*, *glpTQ-glpABC*, *melR-melAB*, *idnDOTR-idnK*, *gntP-uxuAB* and *yfhA-crp*, multiple sites of CRP binding exist even though the regulatory role of each CRP site has not yet been established (Fig. 4C).

## Discussion

Carbon availability in the environment influences the expression pattern of a number of genes in E. coli in various ways. The effector cAMP is a signal generated after sensing the external carbon source availability in the absence of glucose. cAMP-bound active form of CRP is then regulates transcription of a number of genes that are involved in the utilization of non-glucose carbon sources in the absence of glucose [9], [11]–[13], and thus it is an antagonist of catabolite repression [9], [12].

### The whole set of regulation targets of CRP

A number of approaches *in vitro*, *in vivo* and *in silico* have been employed for the identification of regulation targets by cAMP-CRP, and mixed together, a total of 195 target promoters are listed In Regulon DB. Some of these previously known CRP sites were predicted after *in silico* searching for the known CRP-box consensus sequence without experimental confirmation. After Genomic SELEX screening, however, we identified a total of 245 different sequences of CRP binding, and predicted a total of 275 target promoters under the control of CRP. Of the 195 known targets, 92 promoters are included in this list of candidate targets selected at the cut-off level of 4.0 on the SELEX-chip pattern (see Fig. 1). If the rest of 103 CRP targets that are listed in Regulon DB but has not been identified by the Genomic SELEX are added, the total number of CRP targets should increase to at least 378 promoters, leading to about 2-fold sudden increase in the regulation target of CRP (378/195).

More than 50% (103/195) of the CRP targets in Regulon DB was, however, not identified in the Genomic SELEX screening under the conditions herewith employed, but it is noteworthy that the level of consensus sequence conservation is lower for CRP-binding sequences included in these unselected promoters (see Fig. 2). The number of CRP targets should increase by changing the experimental conditions such as that: i) the number of SELEX cycles was reduced to pick up DNA sequences with weak affinity to CRP; or ii) the cut-off level of SELEX-chip pattern was set at lower levels. The number of CRP targets should also increase if CRP bound within intergenic regions between divergent promoters regulates transcription toward both directions.

The marked increase in the numbers of target genes under the control of CRP indicates that the regulation targets by each of a total 300 species of transcription factors in *E. coli* [38], [44] must also be more than those so far identified. In fact, the number of regulation targets of Cra increased 8-fold from 20 to 178 after the Genomic SELEX screening [31]. In the case of PdhR, the regulator for the genes encoding pyruvate dehydrogenase (PDH) multienzyme complex, was found to control the genes for the downstream metabolism, *i.e.*, the respiratory electron transport pathway, including *ndh* (NADH dehydrogenase) and *cyoABCDE* (cytochrome boptype oxidase) [45]. Thus, the Genomic SELEX screening should be a useful experimental screening system for search of the whole set of regulation targets for each transcription factor.

### Regulatory roles of CRP: Regulation of the genes for transport of carbon sources

The most important regulatory role of CRP has been believed to be the control of central metabolism [7], [16]. The genes for glucose metabolism including both glycolysis and gluconeogenesis can be divided into 4 groups: (*a*) the genes for carbon source transporters; (*b*) a large number of genes coding for enzymes participating in the reversible reactions of the central carbon metabolism; (*c*) a small number of the genes coding for rate-controlling enzymes in the carbon metabolism; and (*d*) the genes for side pathways linked to the central carbon metabolism. The group-a genes encoding transporters of carbon sources are organized into more than 40 transcription units, of which more than 30 have been identified to be under the control of CRP [9], [12].

**Figure 5:**
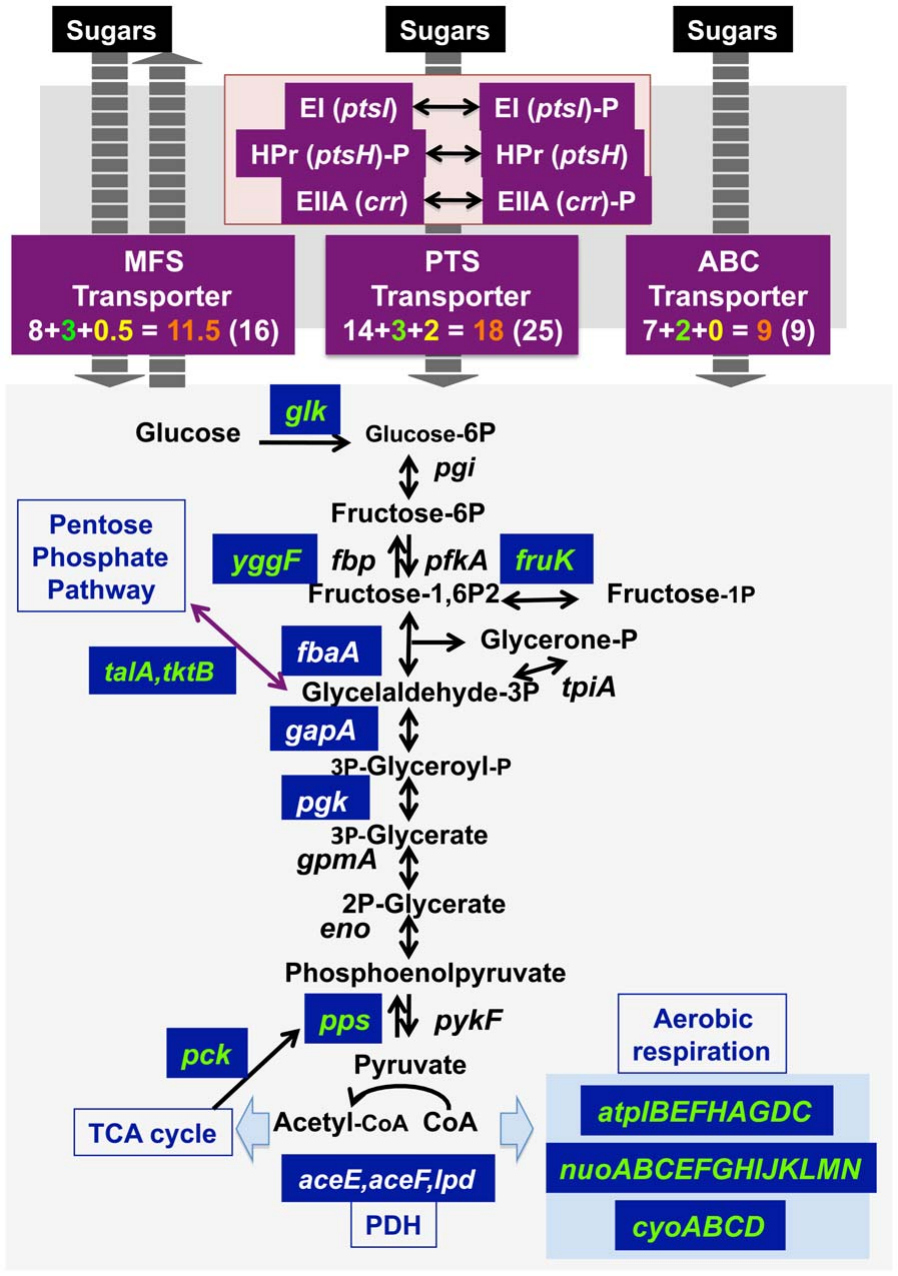
Regulatory roles of CRP. **Upper panel:** The genes under the control of CRP are indicated under either purple background (the genes for sugar transport systems) or blue background (the genes for metabolic enzymes). CRP regulates the majority of genes for three pathways of sugar transport (MFS, PTS and ABC). The number in boxes represent in the order from left to right: The number of CRP target genes listed in Regulon DB (white); the number of genes identified to be regulated by CRP in this study (green); the number of genes predicted to be under the control of CRP in this study (yellow); the total number of genes under the control of CRP (orange). The numbers shown in parenthesis represent total number of genes constituting the respective transport systems, including those not regulated by CRP. **Lower panel:** Most of the genes for the enzymes involved in glycolysis are controlled by Cra [30] while only three genes, *fbaA, gapA* and *pgk*, had been identified as the regulation targets of CRP (shown in while under blue background). In this study, we identified a number of novel targets of CRP (shown in green under blue background). Furthermore a number of the genes involved in the metabolism downstream of glycolysis including PDH pathway and aerobic respiration were found to be the targets of CRP regulation. The number of target 0.5 represents such a particular case as *fbaB*<CRP>*yegT* (see Table S3), in which one (*fbaB*) of the divergent promoters is known under the control of CRP but possible regulation of the opposite promoter (*yegT*) by CRP can not be ruled out.

*E. coli* contains a set of substrate-specific transport systems, which are classified on the basis of molecular composition into PTS (phosphoenolpuruvate: sugar phosphotransferase) [46]–[48], ABC (ATP-binding cassette transporter) [49], MFS systems (major facilitator superfamily) [50] and several unclassified transporters. A total of 25 PTS members have been identified in *E. coli* [46]–[48] (Fig. 5). After Genomic SELEX screening, some additional PTS system genes for sugar transport were found to belong the CRP regulon, including *fruBKA* for fructose [51], [52], *manXYZ* for mannose transport [47], *ptsG* for glucose transport [47], *mtlAD* for mannitol transport [47] and *ptsH* encoding HPr component of PTS [47]. Transcription of *fruAB*, *ptsH* and *pstG* was confirmed to be down-regulated in the crp mutant (see Fig. 3). As a result, almost all the genes for transport of carbon sources are now identified to be under the direct control of cAMP-CRP.

ATP-binding cassette (ABC) transporters belong to the primary transporters, which use the energy released from ATP hydrolysis to drive ions or solutes across membranes. In *E. coli*, ABC transporter consists of import systems that involve the substrate binding protein, transporters of substrates, and outer membrane receptors or porins [53]–[55]. In this study, we identified some ABC-family transporter for carbon sources such as *ugpB* for glycerol-3-phosphate transport [56] and *xylF* for xylose transport [56] as the novel regulation targets of CRP. Including these newly identified targets, all the ABC family transporters for carbon source transport in *E. coli* are now recognized to be all under the control of cAMP-CRP (Fig. 5). Likewise, at least 11 or 12 members of a total of 16 MFS (major facilitator superfamily) transporters [57], [58] were found to belong to the CRP regulon. Taken all these observations together, we conclude that one of the major regulatory roles of CRP is the sorting of transport of carbon sources by controlling the level of substrate-specific transporters within the membranes.

### Regulatory roles of CRP: Regulation of the genes for carbon metabolism

A limited number of the genes encoding the enzymes involved in glycolysis had been found to be regulated by CRP, including *fabA* (fructose-1,6-bisphosphate aldolase), *gapA* (glyceraldehyde-3-phosphate dehydrogenase) and *pgk* (phosphoglycerate kinase) (Fig. 5). In addition, we identified by Genemic SELEX screening that *glk* (glucokinase), *fruK* (1-phosphofructokinase), *yggF* (type II fructose-1,6-bisphosphatase), ppsA (phosphoenolpyruvate synthase), *talA* (transaldolase) and tktB (transketolase II) are under the control of activator cAMP-CRP (Fig. 5). Furthermore the *in vivo* reporter assay indicated that both pgi (glucosephosphate isomerase) and *gpmM* (phosphoglycero mutase III) promoters were activated by CRP while the *fbaB* (class-I fructose-bisphosphate aldolase) promoter was repressed by CRP (Fig. 5; for experimental details see Fig. 3) even though these genes were not detected by Genomic SELEX screening under the experimental conditions and the cut-off level of SELEX-chip pattern herein employed.

The pentose phosphate (PP) pathway, also called hexose monophosphate shunt, is one of the three essential pathways, EMP (Embden-Meyerof-Parnas pathway), PP (pentose-phosphate) and TCA (tri-carboxylic-acid pathway) pathways of the central metabolism. At the entry gate, D-glucose-6P is converted to D-glucono-lactone-6P by Zwf [59], which is metabolized to D-glycraldehyde-3-phosphate by a set of enzymes, Pgi, Gnd, RpiAB, and TktAB, for re-use in the central metabolism [60], [61]. After Genomic SELEX, we found that CRP activates *tktAB* and *talAB* encoding the last step enzymes.

### Regulatory roles of CRP: Regulation of the genes for aerobic respiratory

CRP was found to regulate a number of the genes coding for enzymes involved in the metabolism downstream of glycolysis, including pyruvate dehydrogenase (PDH) complex, and most of the TCA cycle enzymes. Previously we identified by Genomic SELEX screening that PdhR, originally identified as the regulator of genes coding for components of PDH complex, regulates *ndh* encoding NADH dehydrogenase II and *cyoABCDE* encoding the cytochrome *bo*-type oxidate, both together forming the pathway of respiratory electron transport downstream from the PDH cycle [45], [62]. Likewise CRP was found to regulate NADH-ubiquinone oxidoreductase I encoded by the *nuoABCDEFGHIJKLMN* operon, NADH-ubiquinone oxidoreductase II encoded by *ndh*, and the cytochrome *bo* terminal oxidase encoded by the *cyoABCDE* operon. Furthermore the *atpIBFHAGDC* operon coding for the ATPase F1 and F0 complexes was also found to be under the control of CRP (Fig. 5).

### Regulatory roles of CRP: Switching control between glycolysis and gluconeogenesis

Several step reactions of the central carbon metabolism are apparently irreversible, different enzymes being involved in catalysis of forward and backward reactions. These irreversible reactions, sometimes called rate-controlling reactions, are considered to be involved in switching control between glycolysis and gluconeogenesis. There are 4 steps of the irreversible reactions in *E. coli*, two steps at the entry gate of the central metabolism for supply of fructose-1,6-bisphosphate (Fig. 5), *i.e.*, one way reaction of the conversion of glucose to glucose-6-phosphate catalyzed by glucokinase (Glk) [63]; and the synthesis of fructose-1,6-bisphosphate from fructose-6-phosphate catalyzed by 6-phosphofructokinase (PfkA and PfkB) [64] and the reverse reaction catalyzed by four isozymes (Fbp, GlpX, YbhA and YggF) [65]–[68], and two steps at the exit gate from the glycolysis pathway (Fig. 5), *i.e.*, the conversion of phosphoenolpyruvate to pyruvate by two pyruvate kinase isozymes (PykA and PykF) [69], [70] and the reverse reaction catalyzed by phosphoenolpyruvate synthetase (PpsA) [71]; and one way reaction of the synthesis of phosphoenolpyruvate from oxaloacetate by phosphoenolpyruvate carboxykinase (Pck) [72]. When the reactions leading to accumulate fructose-1,6-bisphosphatase are activated, the overall reaction of carbon metabolism is directed toward gluconeogenesis. Among the enzymes involved in these four steps reactions for entry to and exist from the central glycolysis pathway, only one enzyme, GlpX, had been identified as a regulation target of CRP. Here we identified novel targets, Glk, YggF, PpsA, and Pck, indicating that CRP plays a major role in switch control between glycolysis and gluconeogenesis. CRP is activated in the absence of glucose (see below), leading to activate the genes for shifting to gluconeogenesis.

### Transcription factor networks under the control of CRP

**Figure 6:**
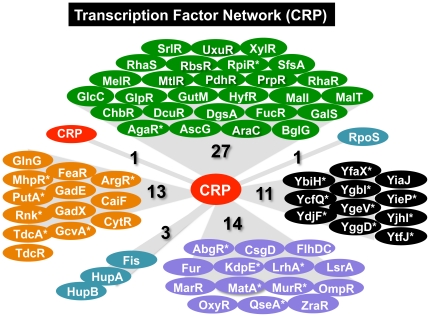
The hierarchy of regulation network of CRP. A total of 70 genes for transcription factors are organized under the control of CRP. Transcription factors are classified on the basis of regulation targets: regulators for carbon metabolism (green background), nitrogen metabolism (orange background), and for stress-response (purple background). Some nucleoid proteins play not only architectural roles but also regulatory roles (blue-green background). A number of genes for uncharacterized transcription factors are under the control of CRP (black background). The set of newly identified CRP targets in this study are shown by asterisk.

Promoters for the genes encoding the key metabolic enzymes and the essential cell architectures are under the control of multiple regulators, each monitoring different environmental conditions or metabolic states [38]. After Genomic SELEX screening, we discovered that a number of genes encoding transcription factors are under the control of CRP (Fig. 6). In the case of CRP, a total of 70 transcription factors are included in the whole set of 349 CRP targets, forming a big hierarchy of transcription factor network. A total of 28 CRP targets including CRP itself are the specific regulators of carbon metabolism (shown under green background in Fig. 6), while 13 are the regulators of nitrogen metabolism (orange background in Fig. 6). Some of this-group regulators such as ArgR and GlnG control a large number of genes (more than 40 by ArgR and more than 50 by GlnG). A total of 14 factors are involved in regulation of the genes for response to external stresses (purple background in Fig. 6). Stationary phase-specific sigma RpoS is also under the control of CRP.

Of the 70 transcription factors under the control of CRP, a total of 24 were newly identified in this study (marked with stars in Fig. 6). One advantage of Genomic SELEX screening system is that regulation targets can be identified even for uncharacterized transcription factors, and thus except for YiaJ, all other transcription factors with unknown functions (black background in Fig. 6) were newly identified to be under the control of CRP.

## Supporting Information

Figure S1 **Location of CRP-binding sites on the regulation target promoters.** The CRP-dependent promoters were predicted by Genomic SELEX screening (Fig. 1 and Tables 1 and 2) and the influence of CRP on some representative promoters was analyzed *in vivo* using the *lacZ* fusion (Fig. 3). The location of CRP-box within 500-bp sequences (between −300 to +200) of these CRP-dependent promoters are indicated by ellipse symbols. Arrows show transcription initiation sites. The numbers represent the distance (bp) from the initiation site of P1 transcription. A: Promoters activated by CRP. B: Promoters repressed by CRP.
(TIF)

Table S1 **Primers used for promoter assay.**
(XLS)

Table S2 **CRP-binding sequences identified by SELEX-clos.**
(XLS)

Table S3 **Regulation targets of cAMP-CRP.**
(XLS)

Table S4 **CRP-binding sequences on regulation target promoters.**
(XLS)
