# Supplementary figures and images for: Novel Roles of cAMP Receptor Protein (CRP) in Regulation of Transport and Metabolism of Carbon Sources
Source: PLoS One. 2011 Jun 1;6(6):e20081. doi: 10.1371/journal.pone.0020081 (PMC3105977; doi:10.1371/journal.pone.0020081)

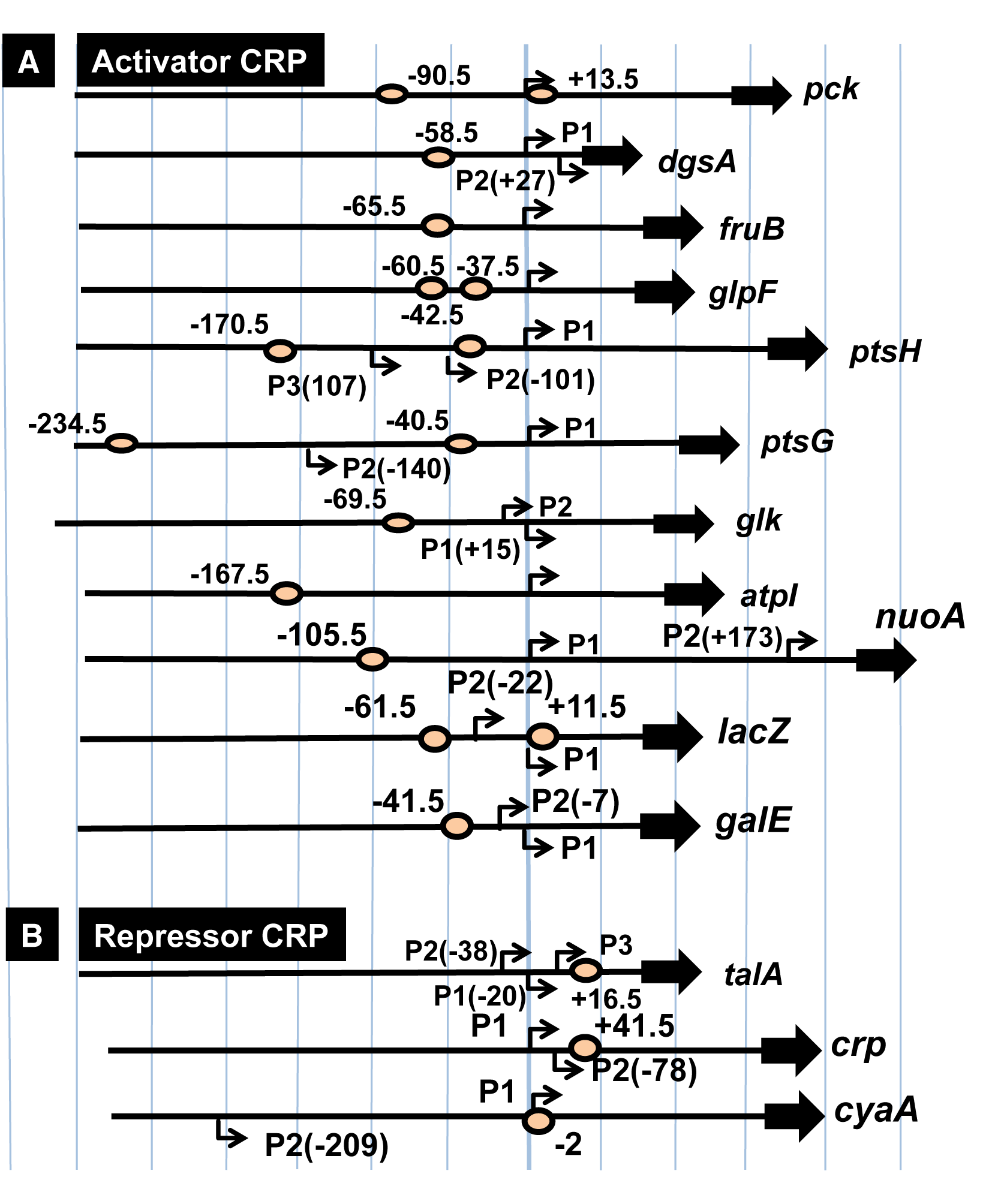

Supplement: Figure S1 — Location of CRP-binding sites on the regulation target promoters. The CRP-dependent promoters were predicted by Genomic SELEX screening (Fig. 1 and Tables 1 and 2) and the influence of CRP on some representative promoters was analyzed in vivo using the lacZ fusion (Fig. 3). The location of CRP-box within 500-bp sequences (between −300 to +200) of these CRP-dependent promoters are indicated by ellipse symbols. Arrows show transcription initiation sites. The numbers represent the distance (bp) from the initiation site of P1 transcription. A: Promoters activated by CRP. B: Promoters repressed by CRP. (TIF) [file pone.0020081.s001.tif]
